# Isosteviol Has Beneficial Effects on Palmitate-Induced α-Cell Dysfunction and Gene Expression

**DOI:** 10.1371/journal.pone.0034361

**Published:** 2012-03-27

**Authors:** Xiaoping Chen, Kjeld Hermansen, Jianzhong Xiao, Sara Kjaergaard Bystrup, Lorraine O'Driscoll, Per Bendix Jeppesen

**Affiliations:** 1 The Department of Endocrinology and Metabolism MEA, Aarhus University Hospital, Aarhus, Denmark; 2 Department of Endocrinology, China-Japan Friendship Hospital, Beijing, China; 3 School of Pharmacy and Pharmaceutical Sciences, Trinity College Dublin, Dublin, Ireland; Universita Magna-Graecia di Catanzaro, Italy

## Abstract

**Background:**

Long-term exposure to high levels of fatty acids impairs insulin secretion and exaggerates glucagon secretion. The aim of this study was to explore if the antihyperglycemic agent, Isosteviol (ISV), is able to counteract palmitate-induced α-cell dysfunction and to influence α-cell gene expression.

**Methodology/Principal Findings:**

Long-term incubation studies with clonal α-TC1–6 cells were performed in the presence of 0.5 mM palmitate with or without ISV. We investigated effects on glucagon secretion, glucagon content, cellular triglyceride (TG) content, cell proliferation, and expression of genes involved in controlling glucagon synthesis, fatty acid metabolism, and insulin signal transduction. Furthermore, we studied effects of ISV on palmitate-induced glucagon secretion from isolated mouse islets. Culturing α-cells for 72-h with 0.5 mM palmitate in the presence of 18 mM glucose resulted in a 56% (*p*<0.01) increase in glucagon secretion. Concomitantly, the TG content of α-cells increased by 78% (*p*<0.01) and cell proliferation decreased by 19% (*p*<0.05). At 18 mM glucose, ISV (10^−8^ and 10^−6^ M) reduced palmitate-stimulated glucagon release by 27% (*p*<0.05) and 27% (p<0.05), respectively. ISV (10^−6^ M) also counteracted the palmitate-induced hypersecretion of glucagon in mouse islets. ISV (10^−6^ M) reduced α-TC1–6 cell proliferation rate by 25% (*p*<0.05), but ISV (10^−8^ and 10^−6^ M) had no effect on TG content in the presence of palmitate. Palmitate (0.5 mM) increased Pcsk2 (*p*<0.001), Irs2 (*p*<0.001), Fasn (*p*<0.001), Srebf2 (*p*<0.001), Acaca (*p*<0.01), Pax6 (*p*<0.05) and Gcg mRNA expression (*p*<0.05). ISV significantly (*p*<0.05) up-regulated Insr, Irs1, Irs2, Pik3r1 and Akt1 gene expression in the presence of palmitate.

**Conclusions/Significance:**

ISV counteracts α-cell hypersecretion and apparently contributes to changes in expression of key genes resulting from long-term exposure to palmitate. ISV apparently acts as a glucagonostatic drug with potential as a new anti-diabetic drug for the treatment of type 2 diabetes.

## Introduction

Nearly four decades ago, the bihormonal-abnormality hypothesis [1] established the essential role for glucagon in diabetes, by highlighting the contributions of deficient insulin secretion, development of insulin resistance and increased glucagon secretion to the hyperglycemic state in type 2 diabetes. Glucagon, secreted from the pancreatic α-cells, plays a critical role in the regulation of glycemia [2]. This hormone counteracts hypoglycemia and opposes insulin actions by stimulating hepatic glucose synthesis and mobilization, thereby increasing blood glucose concentrations [3]; a large body of evidence shows that hyperglucagonemia is essential to the maintenance of increased rates of hepatic glucose output in type 2 diabetes [4], [5].

Elevated levels of free fatty acid (FFA) are apparent in patients with diabetes and prediabetes, suggesting that FFAs may be involved in the pathophysiology of type 2 diabetes [6]–[8]. The information on the effects of FFA on α-cells is sparse. It was recently demonstrated that palmitate has an acute stimulating effect on glucagon secretion from murine and rat islets [9], [10]. We have subsequently clarified that the acute fatty acid-induced stimulation of glucagon secretion depends on the chain length, spatial configuration, and degree of unsaturation of fatty acids [11]. Interestingly, long-term exposure of clonal α-cells to palmitate caused enhanced glucagon secretion and TG accumulation in a time- and dose-dependent manner, while α-cell proliferation was inhibited [12]. In agreement with this, long-term exposure of rat islets to fatty acids elicited marked increase in glucagon release, and decreased glucagon content; both apparently without changing the glucagon gene expression [13].

However, the cellular and molecular mechanisms whereby FFAs modulate the coupling of α-cell stimulus secretion remain an enigma [14]. Understanding the mechanism(s) of FFAs action is of potential importance for the development of novel, efficient therapies in diabetes. The potential of reducing glucagon secretion (*e.g.* using GLP-1) or inhibiting glucagon action (*e.g.* with glucagon receptor antagonists) have received much attention as therapeutic strategies for the treatment of excess glucose production in type 2 diabetic patients [15], [16].

Stevioside, a diterpene glycoside isolated from leaves of the plant Stevia Rebaudiana Bertoni, represents a new class of insulin secretagogues chemically unrelated to previous oral antidiabetic drugs, such as sulphonylureas. Interestingly, we have shown that stevioside is able to counteract α-cells hypersecretion caused by palmitate and to enhance the expression of genes involved in fatty acid metabolism [17]. Furthermore, we have demonstrated that the stevioside derivate, ISV, has a higher bioavailability and a higher potency on islet cell function and glycemic control when compared to Stevioside, with the same low toxicity as Stevioside [18].

The present study was, thus, designed to elucidate the potential role of ISV on α-cell function and its effects on expression of specific genes following long-term exposure to palmitate. Our findings provide evidence that ISV decreases palmitate-induced hyperglucagonemia in both the mouse pancreatic α-cell line, αTC1–6 cells, and in isolated mice islets. ISV concomitantly restores the palmitate-induced impairment of insulin secretion from islets, affects intracellular insulin signal and α-cell proliferation.

## Results

### Impacts of ISV on palmitate-induced glucagon and insulin secretion from isolated murine islets

**Figure 1:**
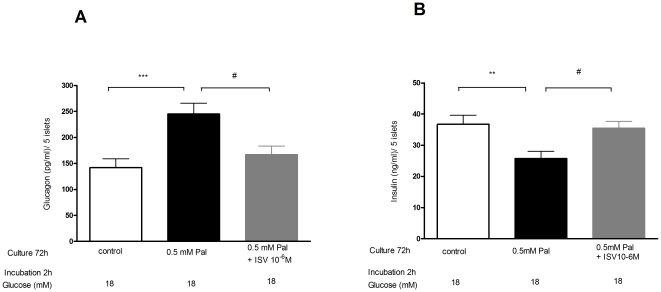
Anti-lipotoxicity and anti-glucotoxicity effects of isosteviol (ISV) in isolated mice islets. Islets were incubated at 11.1 mM glucose in the presence of 0 mM, 0.5 mM palmitate (Pal) and 0.5 mM Pal with 10^−6^ M ISV for 72 h. Glucagon (**A**) and insulin (**B**) secretion were measured by incubation of 5 islets with medium containing 18 mM glucose for 2 h. Data is presented as mean ± SEM (n = 18 samples each group from 3 separate experiments). *P<0.05, **P<0.01, ***P<0.001 vs. control. ^#^P<0.05 vs. 0.5 mM Pal.

To evaluate the impacts of ISV on the secretion of glucagon and insulin from islets in the presence of palmitate, the isolated mouse islets were cultured in medium supplemented with 0 mM palmitate, 0.5 mM palmitate or 0.5 mM palmitate plus ISV (10^−6^ M). To mimic hyperglycemia characteristic of the diabetic state, the islets were incubated in fresh KRB medium containing 18 mM glucose for 2 h after incubating for 72 h in conditions as described above. It was found that pre-treatment with palmitate substantially increased glucose induced-glucagon secretion by 73% (*p*<0.001; Fig. 1A) and suppressed glucose-stimulated insulin secretion by 30% (*p*<0.01; Fig. 1B). In contrast, pre-treatment with ISV (10^−6^ M) significantly suppressed palmitate-induced hyper-secretion of glucagon by 32% (*p*<0.05; Fig. 1A), while it ameliorated palmitate-suppressed insulin secretion by 38% (*p*<0.05; Fig. 1B).

### Effects of ISV short-term pretreatment on glucagon secretion from palmitate-treated α- TC1–6 cells

**Figure 2:**
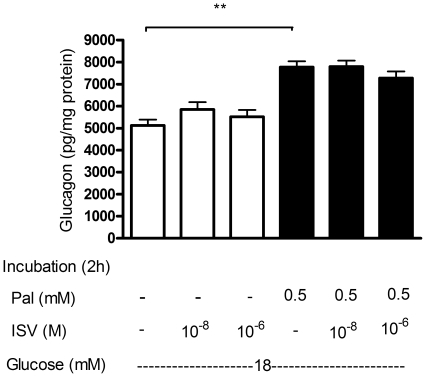
Effects of short-term pretreatment with 10^−8^ M and 10^−6^ M ISV on glucagon secretion from palmitate-treated α-TC1–6 cells. We studied glucagon secretion following 2-hour incubation with or without 0.5 mM palmitate in the presence or absence of 10^−8^ M and 10^−6^ M ISV in medium containing 18.0 mM glucose. Data is presented as mean ± SEM of 24 samples per group from three independent experiments. One-way ANOVA post hoc analyses: **P<0.01 vs. 18 mM glucose alone.

After 2 h incubation with or without 0.5 mM palmitate in the presence or absence of ISV (10^−8^ M and 10^−6^ M) at high glucose (18 mM glucose), 0.5 mM palmitate increased glucagon secretion by 52% (*p*<0.01; Fig. 2). Short-term pretreatment with ISV (10^−8^ M and 10^−6^ M) had no effect on glucagon secretion by palmitate-treated cells (Fig. 2). Glucagon secretion was unchanged by ISV per se (Fig. 2).

### Inhibitory effects of long-term pretreatment with ISV on palmitate-induced glucagon secretion

**Figure 3:**
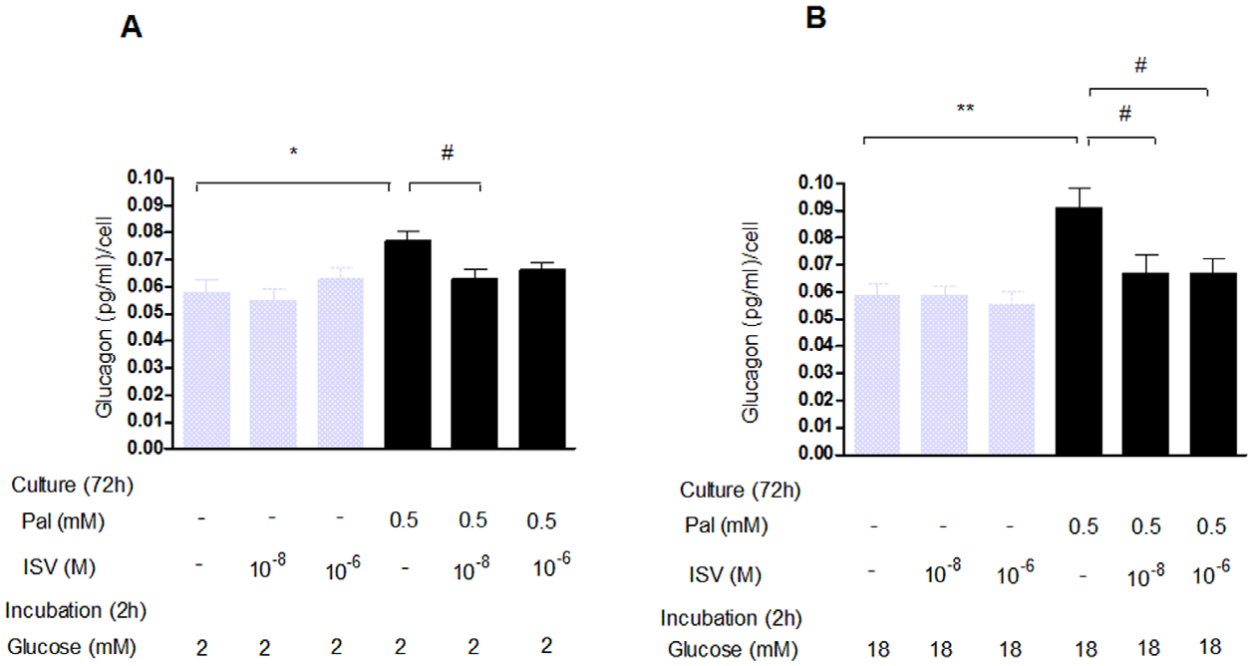
The inhibitory effects of long-term pre-treatment (72 h) with ISV on palmitate-induced glucagon secretion. α-TC1–6 cells were incubated, for 72 h, with or without 0.5 mM palmitate (Pal) (gray bars, without Pal; black bars, with Pal) in the presence or absence of ISV (10^−8^ to 10^−6^ M). The cells were then incubated in medium containing 2 mM glucose (**A**) or 18 mM glucose (**B**) for 2 h. Data is presented as mean ± SEM of 18–20 samples per group from three independent experiments. One-way ANOVA post hoc analyses: *P<0.05, **P<0.01 compared with glucose alone. ^#^P<0.05 compared with Pal alone.

Figure 3 demonstrates the effect of 72 h culture with ISV (10^−8^ and 10^−6^ M), without or with 0.5 mM palmitate, on glucagon secretion in the presence of 2 mM glucose (Fig. 3A) or 18 mM glucose (Fig. 3B), respectively. Palmitate resulted in a significant stimulation of glucagon secretion both in the presence of low (2 mM) and high (18 mM) glucose by 34% (*p*<0.05) and 56% (*p*<0.01), respectively. ISV alone (10^−8^ and 10^−6^ M) had no effect on glucagon secretion. However, in the presence of 0.5 mM palmitate, the palmitate-induced glucagon release was inhibited by 18% (*p*<0.05) in the presence of 10^−8^ M ISV at 2 mM glucose. Furthermore, at 18 mM glucose, glucagon secretion was decreased by 27% (*p*<0.05) and 27% (*p*<0.05), respectively, by 10^−8^ M and 10^−6^ M ISV.

### Influence of palmitate and ISV on glucagon content

**Figure 4:**
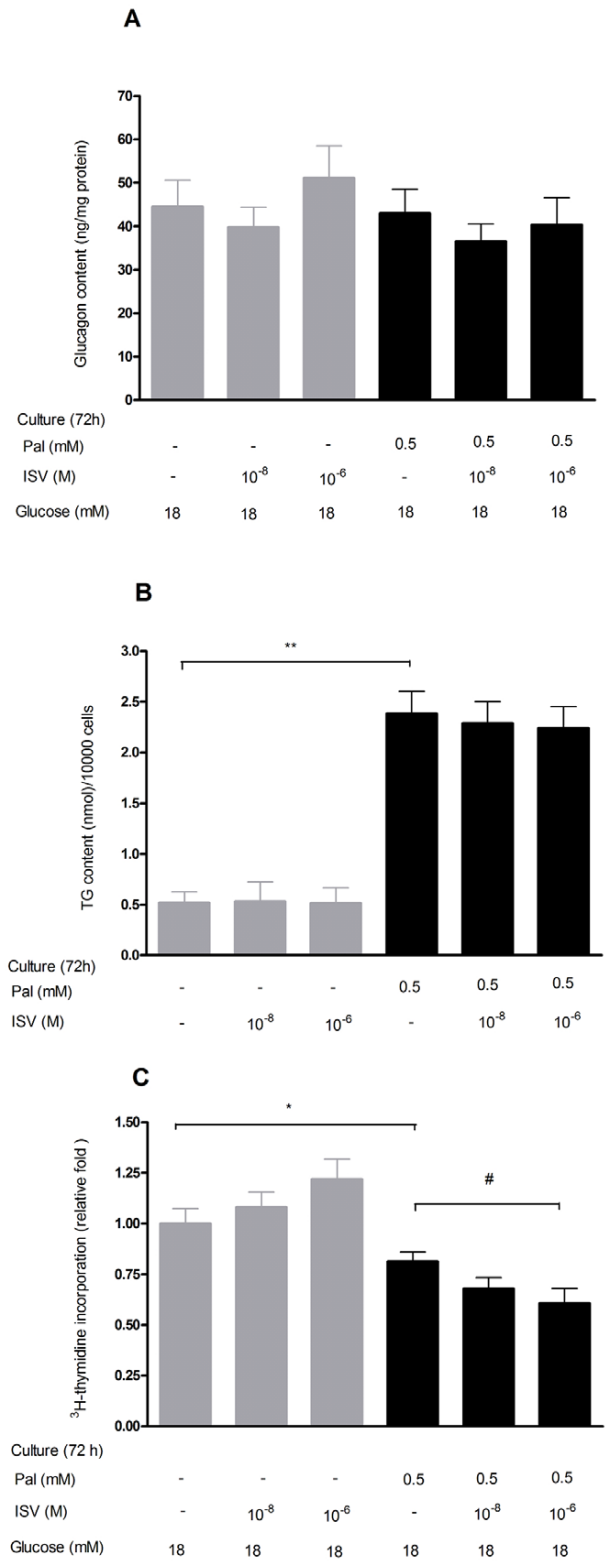
Impact of ISV on glucagon content, TG content, and ^3^H-thymidine incorporation in α-TC1–6 cells. After 72 h pre-treatment with or without 0.5 mM palmitate (Pal) in the presence or absence of ISV 10^−8^ M and 10^−6^ M; illustrating glucagon content, TG content and ^3^H-thymidine incorporation in α-TC1–6 cells. Data is presented as mean ± SEM; n = 10–12 per group in glucagon content (**A**) and n = 21–24 per group in TG content (**B**), whereas data in ^3^H-thymidine incorporation (**C**) are expressed as a relative ratio compare to control group of n = 30 per group from three independent experiments. *P<0.05, **P<0.01 vs. control. ^#^P<0.05 vs. Pal.

As illustrated at Figure 4A, we found a tendency toward reduced glucagon content after 72 h culture with palmitate (0.5 mM), however, this did not reach statistical significance. ISV had no impact on glucagon content after 72 h culture with 0.5 mM palmitate.

### Impact of palmitate and ISV on TG content

The effect of palmitate and ISV on TG content in α-TC1–6 cells cultured for 72 h is shown in Figure 4B. Palmitate significantly increased intracellular TG content by 78% (*p*<0.01), however, the addition of ISV (10^−8^ M, 10^−6^ M) in the presence of palmitate had no impact on the TG content.

### Impact of ISV on palmitate-induced changes on ^3^H-thymidine incorporation

To determine whether the inhibitory effect of ISV on palmitate-induced glucagon secretion could be mediated through cell proliferation, ^3^H-thymidine incorporation was used to assess cell proliferation. Within 72 h at 18 mM glucose, 0.5 mM palmitate reduced the ^3^H-thymidine incorporation by 19% compared with glucose alone (*p*<0.05). At 0.5 mM palmitate, 10^−6^ M ISV caused a 25% (*p*<0.05; Fig. 4C) decrease in ^3^H-thymidine incorporation.

### Effects of palmitate and ISV on gene expression

**Figure 5:**
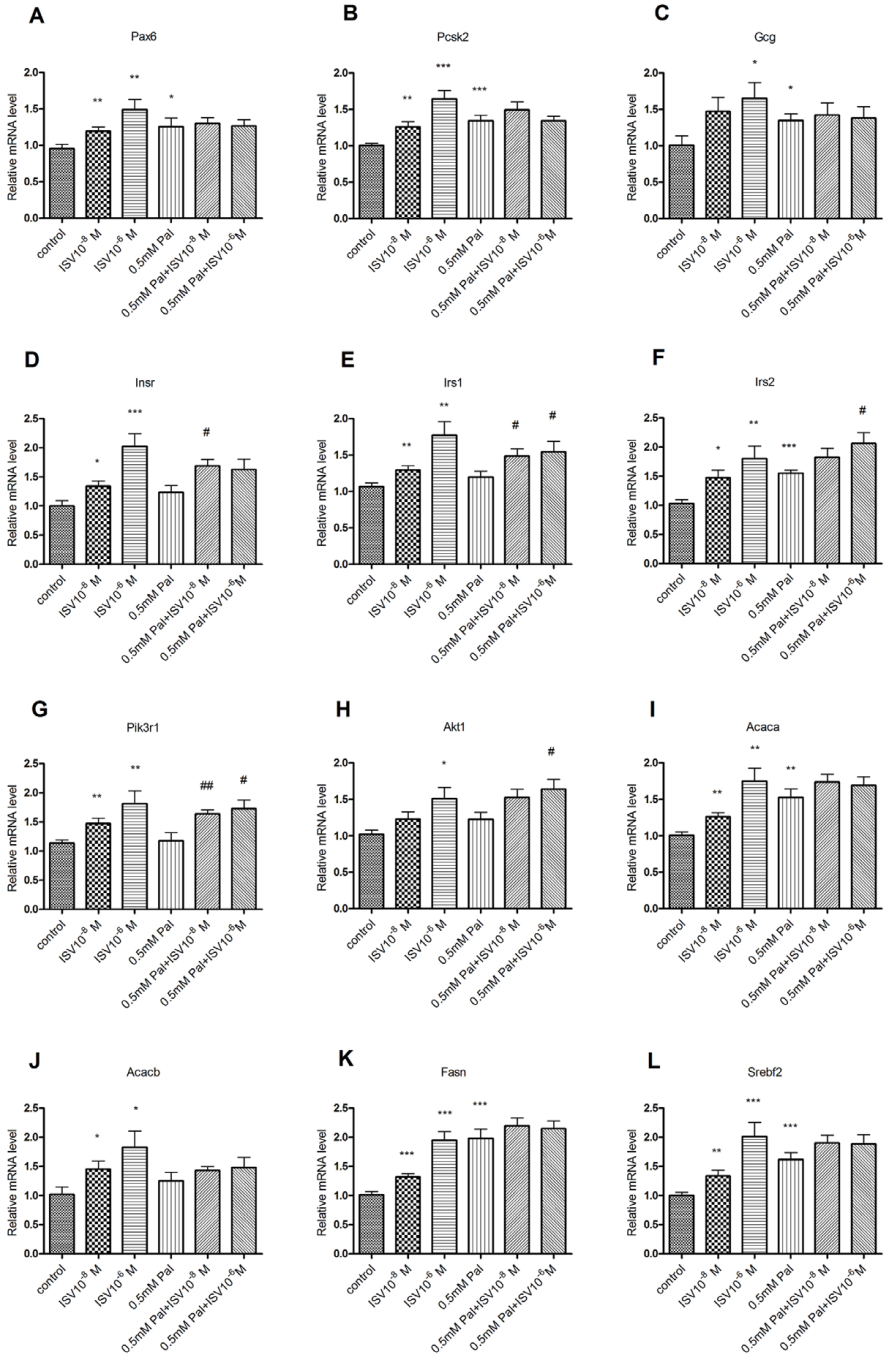
Effects of palmitate and ISV on gene expression. After 72 h of treatment with or without 0.5 mM palmitate (Pal) in the presence or absence of ISV 10^−8^ M and 10^−6^ M, gene expression levels in α-TC1–6 cells were determined and normalized to Hprt. Data is expressed as relative level to control (n = 10–12 samples in each group, RNA extracted from three independent experiments). *P<0.05, **P<0.01, *** P<0.001 vs. control. ^#^P<0.05, ^##^P<0.01 vs. 0.5 mM Pal.

To gain further insight into the counteractive effect of ISV on palmitate-induced hypersecretion of glucagon, the expression of a few selected genes was examined in α-TC1–6 cells (Fig. 5). Palmitate alone increased the expression of proprotein convertase subtilisin/kexin type 2 (Pcsk2, *p*<0.001), insulin receptor substrate 2 (Irs2, *p*<0.001), fatty acid synthase (Fasn, *p*<0.001), sterol regulatory element binding factor 2 (Srebf2, *p*<0.001), acetyl-Coenzyme A carboxylase alpha (Acaca/ACC1, *p*<0.01), paired box gene 6 (Pax6, *p*<0.05) and glucagon (Gcg, *p*<0.05). ISV alone significantly increased expression of all the selected genes, in the absence of palmitate (10^−8^ M, *p*<0.05; 10^−6^ M, *p*<0.001). Compared with cells cultured with palmitate alone, cells cultured with ISV (10^−8^ M) had a significantly higher expression of insulin receptor (Insr), insulin receptor substrate 1 (Irs1), and phosphatidylinositol 3-kinase, regulatory subunit, polypeptide 1 (p85 alpha) (Pik3r1) genes in the presence of palmitate, while cells cultured with a higher concentration of ISV (10^−6^ M) displayed significantly higher expression levels of Irs1, Irs2, Pik3r1 and thymoma viral proto-oncogene 1 (Akt1) gene in the presence of palmitate. Glucagon receptor (Gcgr) gene expression was undetected in α-TC1–6 cells (unpublished data).

### Effects of α-cells and β-cells interaction on gene expression

**Figure 6:**
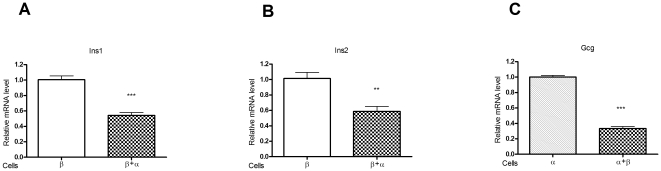
Effects of α-TC1–6 cells and or MIN6 cells on gene expression. Ins1 (**A**) and Ins2 (**B**) expression in MIN6 cells when cultured, for 72 hours, alone or in combination with α-TC1–6 cells. Gcg (**C**) expression in α-TC1–6 cells cultured, for 72 hours, alone or in combination with MIN6 cells. All data are expressed as relative level to control (n = 6 samples per group). **P<0.01, ***P<0.001.

To investigate the interaction between α-cells and β-cells, α-TC1–6 cells and MIN6 cells were co-cultured. The effects of co-culture on the expression of the insulin1 (Ins1) and insulin2 (Ins2) genes (specific to β-cells) and on the expression of Gcg gene (specific to α-cells) were examined. The co-culturing was found to result in significantly decreased expression levels of Ins1 (*p*<0.001, Fig. 6A), Ins2 (*p*<0.01, Fig. 6B) and Gcg (*p*<0.001, Fig. 6C), as compared with the independently culture of β-cells and α-cells.

## Discussion

Here we have demonstrated that incubation of α-TC1–6 cells for 72 h with 0.5 mM palmitate significantly increased their glucagon secretion. Concomitantly, at high glucose concentrations the TG content was increased, while α-TC1–6 cell proliferation was decreased. In addition, their overall glucagon content was unchanged. Interestingly, ISV (10^−8^ and 10^−6^ M) counteracted the palmitate-induced glucagon secretion. Short-term incubation of α-TC1–6 cells with 0.5 mM palmitate resulted in enhanced glucagon secretion under conditions of high glucose, while ISV (10^−8^ M and 10^−6^ M) had no effect on glucagon secretion during short-term incubation. As expected, exposure of murine islets to 0.5 mM palmitate for 72 h resulted in decreased glucose-stimulated insulin secretion (GSIS) and glucagon hypersecretion. Our results show that ISV (10^−6^ M) simultaneously counteracts the decrease in GSIS and abolishes glucagon hypersecretion from murine islets. ISV (10^−6^ M) concomitantly reduces α-cells proliferation. ISV did not change the TG or glucagon content of α-cells in the presence of 0.5 mM palmitate. The expression of Pax6, Pcsk2, Gcg, Irs2, Acaca, Fasn and Srebf2 were up-regulated during lipotoxicity, while acetyl-Coenzyme A carboxylase beta (Acacb/ACC2), Insr, Irs1, Pik3r1 and Akt1 remained unchanged. Interestingly, ISV (10^−8^, 10^−6^ M) significantly increased the expression of Insr, Irs1, Irs2, Pik3r1 and Akt1 during lipotoxicity. However, expression of Pax6, Pcsk2, Gcg, Acaca, Acacb, Fasn and Srebf2 genes were not affected by these conditions. Gcgr expression was undetected in α-TC1–6 cells but was detected in liver tissue from mice, using the same Gcgr assay (unpublished data). Whether or not it is expressed in normal α-cells has yet to be determined.

We previously demonstrated that, during lipotoxicity, the glycosylated form of ISV, Stevioside, counteracts the impaired insulin secretion from both rat islets and INS-1E cells [19]. Stevioside was able to lower blood glucose in type 2 diabetic subjects [20] and in a non-obese animal model of type 2 diabetes *i.e.* the Goto Kakizaki rat [21] by both increasing insulin release and suppressing glucagon levels [22]. ISV has a critical glucose-dependent effect on insulin secretion and has proven to be more potent than other ISV derivates found in the plant. ISV improves blood glucose and insulin sensitivity and upregulates the expression of key beta-cell genes *e.g.* Irs1and Pax6 in type 2 diabetic KKAy mice [18].

It is well established that glucagon secretion is regulated by multiple complex factors including circulating glucose, FFAs, amino acids, somatostatin, products secreted by β-cells, including insulin, γ-aminobutyric acid (GABA), zinc ions and glutamate, the central and autonomic nervous systems and several hormones [14]. However, among these factors, insulin, undoubtedly, plays a critical role in glucagon secretion [23]. Several *in vivo* and *in vitro* studies have shown that insulin is a major paracrine glucagon suppressor [24]–[26], and that an increase in the insulin concentration suppresses glucagon secretion [27]. Our data also showed a substantial decrease in Gcg gene expression in α-cells when co-cultured with β-cells. In fact, insulin inhibits glucagon secretion via several pathways. Insulin, in a phosphatidylinositol-3 (PI-3) kinase-dependent manner, stimulates mouse islet α- and β-cell K_ATP_ channel opening by decreasing their sensitivity to ATP block [28]. Furthermore, insulin increases K_ATP_ channel activity in isolated rat α-cells via membrane hyperpolarization [24]. In addition to the effects on K_ATP_ channels, insulin has been reported to activate A-type GABA receptors in the α-cells by receptor translocation via activation of Akt *i.e.* a critical downstream effector of PI3K. This leads to membrane hyperpolarization in the α-cells and thus suppression of glucagon secretion [29]. Insulin also acts in the ventromedial hypothalamus *via* activation of the PI3K signalling pathway to influence and possibly fine-tune- the release of pancreatic glucagon, under both hypoglycemic and fasting conditions [30].

To further elucidate potential mechanisms whereby ISV regulates glucagon secretion, we utilised the clonal pancreatic α-cell line, α-TC1–6 cells. These cells show characteristics of native islet α-cells, but do not produce insulin and somatostatin at detectable levels [31]. It is our understanding that this is the first study reporting effects of ISV on glucagon secretion from palmitate-exposed α-cells. Here we demonstrated that short-term exposure of α-cells to elevated palmitate induced glucagon hypersecretion. Although short-term exposure to ISV (10^−8^ M and 10^−6^ M) had no effect on glucagon secretion, long-term incubation with ISV (10^−8^ M and 10^−6^ M) resulted in a significant reduction in glucagon hypersecretion elicited by palmitate. It is noteworthy that ISV (10^−8^ M and 10^−6^ M), in the presence of palmitate, also enhanced Insr, Irs1, Irs2, Pik3r1 and Akt1 expression. The highest ISV dose (10^−6^ M) elicited more pronounced changes in gene expression than the lower dose in the absence of palmitate (Fig. 5A–5L). However, when palmitate was added, the differences in gene expression at various ISV doses faded. The reason for this is not known.

In addition to failure of β cells to secrete insulin, the α-cell dysfunction in type 2 diabetes seems to result from insulin resistance of these glucagon-producing cells [27]. Insr and its downstream signaling proteins are abundantly expressed in pancreatic α-cell lines [32]. The high level of Insr expression by α-cells suggests that these cells are also important sites of insulin action [24]. In studies of glucagon-secreting InR1G cells and α-TC6 cells, Insr knockdown (using siRNA) not only enhanced glucagon secretion [33], [34], but also reduced cell growth [34]. Adult male α-cell specific Insr knockout (αIRKO) mice have been shown to exhibit mild glucose intolerance, hyperglycemic and hyperglucagonemia in the fed state and enhanced glucagon secretion in response to L-arginine stimulation which led to increased glucose release from the liver. Moreover, αIRKO mice displayed an abnormal glucagon response to hypoglycemia [33]. In α-cells, the activation of Insr/Irs1/Pi3k/Akt may eventually lead to membrane hyperpolarization, regulate K-channel sensitivity to ATP and reduce glucagon release [35]. Extended exposure of α-cells to insulin has been shown to result in Insr downregulation and loss of insulin-regulated inhibition of glucagon gene transcription [36]. Together, these data indicate a substantial role for intra-islet insulin signaling in the regulation of α-cell function. α-cell studies are consistent with a large number of studies of insulin action on its classic target tissues. Insulin is known to regulate pancreatic β-cell function through the activation of cell surface Insr, phosphorylation of Irs1 and Irs2, and activation of Pi3k [37]. Piro S, et al. [38] recently reported that palmitate affected Insr phosphorylation and intracellular insulin signalling in α-TC1–6 cells, while insulin-stimulated phosphorylation of Insr, Irs-1, Pi3K and Akt were greatly reduced under the same conditions. In α-cells, insulin has been reported to inhibit glucagon release and Gcg gene expression, through phosphorylation of Irs1 and activation of Pi3k [39].

Prohormone convertase 2 (PC2, also referred to as Pcsk2) is the enzyme responsible for processing proglucagon to the active glucagon in pancreatic α-cells [40]. Mice deficient in PC2 have little or no mature circulating glucagon [41]. Pax6, is an important gene for glucagon biosynthesis. It controls some key genes involved in Gcg gene transcription, proglucagon processing enzyme PC2 [42], [43], and α cell differentiation [42]. Therefore, we further explored whether ISV affects Gcg gene transcription in the presence of palmitate. Pax6, Pcsk2 and Gcg gene expression levels were found to be higher in α-TC1–6 cells cultured with palmitate. A similar observation has previously been reported [38]. No changes were found in expression levels of Pax6, Pcsk2 and Gcg with ISV in the presence of palmitate. Although ISV alone increased expression of Pax6, Pcsk2 and Gcg genes related to glucagon biosynthesis, ISV did not alter glucagon secretion. This may be due to upregulation of other key regulatory genes such as Insr, Irs1, Irs2, Pik3r1 and Akt1 which inhibit glucagon secretion. The diverging actions observed may have balanced out each other leading to no net change in the secretion of glucagon in the presence of ISV alone (Fig. 3).

Besides the regulation of glucagon secretion, α-cell growth and survival are of increasing interest in diabetes research. Liu *et al.* reported that elevated plasma glucose levels in diabetic mice is accompanied by increased pancreatic α-cell numbers [44]. We have established that ISV inhibited α-cell proliferation in the presence of 0.5 mM palmitate. Mizushina *et al.* reported that ISV inhibited human cancer cell growth [45]. The precise mechanisms responsible for these alterations remain to be clarified. It is puzzling that palmitate also decrease α-cell proliferation, which may be due to lipotoxicity [12].

In the present study we demonstrated that long-term exposure to palmitate resulted in TG accumulation in α-cells and increased expression of genes that are involved in fatty acid synthesis and oxidation *i.e.* Fasn, Acaca and Srebf2. ISV was found not to reduce TG content and/or alter the expression of fatty acid metabolism-related genes in the presence of palmitate. Surprisingly, we found no change in the glucagon content of α-TC1–6 cells in presence of palmitate. This is, however, in line with Hong et al. [17] who also found no increase in glucagon content in response to Stevioside *i.e.* the glycosylated form of ISV.

In summary, our study demonstrates that chronic exposure to palmitate inhibits insulin secretion, increases glucagon secretion from murine islets and from α-TC1–6 cells, while increasing Pax6, Pcsk2 and Gcg gene expression and TG accumulation. The inhibitory effect of ISV on excess glucagon secretion from murine islets may be due to augmentation of GSIS, which suppresses glucagon secretion. Evidence suggests that the effect of ISV on α-TC1–6 cells may be related to enhanced expression of key regulatory genes involved in insulin signal transduction pathways, thereby increasing insulin sensitivity in α-cells, and concomitantly counteracting hyperglucagonemia. As ISV possesses insulinotropic and glucagonostatic effects, it is likely that ISV may serve as a valuable new therapeutic agent in type 2 diabetes. To further confirm ISV antihyperglycemic effects, clinical trials including consenting people with type 2 diabetes are now warranted.

## Materials and Methods

### Reagents and buffers/solutions

#### Agent preparation

All chemicals were purchased from Sigma-Aldrich (Brøndy, Denmark) if not stated otherwise.

Isosteviol (ISV) (ent-16-ketobeyeran-19-oic acid; Wako Pure Chemical Industries, Osaka, Japan) was added to the medium from a stock solution (10^−3^ M) prepared in 99% ethanol.

50 mmol/l palmitate: The solution was prepared as previously described [17]. Palmitate (Sigma, St. Louis, Mo) was prepared by dissolving and heating equal molar amounts of NaOH and fatty acids, supplemented with distilled water, to obtain a 500 mM concentration. It was further diluted with 5% bovine serum albumin (BSA) (fatty acid free, Sigma) to 50 mM fatty acid. The stock solution was stored at −20°C.

Modified Krebs-Ringer Buffer (M-KRB): 125 mM NaCl, 5.9 mM KCl, 1.2 mM MgCl_2_, 1.28 mM CaCl_2_, 5.0 mM NaHCO_3_, 25 mM HEPES (pH 7.4).

SYTO 24 solution: 5 mM SYTO 24 green fluorescent nucleic acid stain (molecular probes, Invitrogen, Oregon, USA) was dissolved in dimethyl sulfoxside ≥99.9% to a final concentration of 0.01 mM.

### Experimental animals

Adult female Naval Medical Research Institute (NMRI) mice (Bomholtgaard Breeding and Research Center, Ry, Denmark) were maintained on a 12-h light/dark cycle and had free access to water and an ordinary laboratory chow diet. At the time of experiments, they had similar body weights (31–38 g). This study was approved by the Danish Council for Animal Experiments (2010/561-1805).

### Islet Isolation

Islets were isolated by the collagenase digestion technique [46]. In brief, the animals were anaesthetized with pentobarbital (50 mg/kg intraperitoneally) and midline laparotomy was performed. The pancreas was retrogradely filled with 3 ml ice-cold Hanks balanced salt solution (HBSS) (Sigma Chemical, St Louis, MO, USA) supplemented with 0.3 mg/ml collagenase P (Boehringer Mannheim, Mannheim, Germany). HPSS and collagenase P was filter-sterilized before use. The pancreas was subsequently removed and incubated for 19 min at 37°C in a water-bath, and rinsed with ice-cold HBSS. The islets were subsequently handpicked under a stereomicroscope. The islets were then incubated overnight at 37°C and 95% normal atmosphere/5% CO_2_ in 10 ml RPMI 1640 containing 11.1 mmol/l glucose supplemented with 10% fetal bovine serum (FBS), 2.06 mM L-glutamine, 100 IU/ml penicillin G and 100 µg/ml streptomycin (all from GIBCO BRL, Paisley, UK). In each experiment, islets were obtained from 8–10 mice to compensate for inter-individual differences.

### Islet insulin and glucagon secretion studies

This part of the studies aimed to determine whether ISV could prevent lipotoxicity caused by palmitate in mice islets. After overnight culture, the islets were incubated in 4-well plates (NUNC) with fresh medium. Thus, ten islets were incubated in 1 ml RPMI 1640 containing 11.1 mM glucose in the absence or presence of 0.5 mM palmitate, and with 0.5 mM palmitate plus ISV (10^−6^ M). Palmitate solution (0.5 mM) was freshly prepared before each experiment.

After 72 h culture, the islets were rinsed once with M-KRB supplemented with 11.1 mM glucose and 0.5% BSA (fatty acid free, BSA from Roche, Mannheim, Germany). Consequently, batches of five islets were handpicked and incubated in a 37°C water bath with 250 µl of M-KRB containing 0.5% BSA with 18 mM glucose. After 2 h, 150 µl of incubation medium from five islets was collected on ice and frozen for the subsequent analysis of glucagon and insulin secretion. The results of glucagon and insulin were expressed as pg/ml/5 islets, and ng/ml/5 islets, respectively. Glucagon and insulin were analysed by radioimmunoassay (detailed below).

### α-TC1–6 cell and culture conditions

The α-TC1 cell line was derived from an adenoma developed in transgenic mice expressing the SV40 large T-antigen oncogene [31]. The α-TC1 subclone 6 (α-TC1–6 cells, courtesy of Prof. Shimon Efrat, Tel Aviv University, Israel) cells between passages 16 and 49 were grown in Dulbecco's Modified Eagle's Medium (DMEM) (GIBCO-BRL, Paisley, UK) containing 18 mM glucose and 10% FBS (GIBCO-BRL) at 37°C in a humidified (5% CO_2_, 95% air) atmosphere. Cells were passaged once a week and replaced with fresh medium twice weekly. These were seeded at 0.25×10^6^ cells/well (24-well plate) and 0.75×10^6^ cells/well (6-well plate) 24 h before an assay.

### Glucagon secretion from α-TC1–6 cells

#### Short-term incubation

α-TC1–6 cells were pre-incubated, for 15 min, in Krebs-Ringer buffer (KRB) and 0.5% BSA (pH 7.4) and were subsequently incubated for 2 h with KRB containing 18 mM glucose in the presence or absence of 10^−8^ M and 10^−6^ M ISV, 0.5% BSA, 0.5 mM palmitate with or without 10^−8^ M and 10^−6^ M ISV at 18 mM glucose. Subsequently, supernatants (300 µl each) were collected, centrifuged, and 200 µl supernatants were kept frozen at −20°C for glucagon analysis.

### Protein assay

After the acute secretion study, the medium was removed and cells were lysed in 0.1 M NaOH (Sigma). Intracellular protein was measured by Bio-Rad detergent compatible protein assay kit (Bio-Rad Laboratories, Hercules, CA). The glucagon levels in short-term α-TC1–6 cells incubation were adjusted to protein concentration.

### Long-term incubation

α-TC1–6 cells were incubated for 72 h at 37°C in DMEM with 18 mM glucose in the presence or absence of 0.5 mM palmitate (Sigma Chemical, St. Louis, MO), 0.5% BSA (fatty acid free; Roche, Mannheim, Germany) with or without 10^−8^ to 10^−6^ M ISV. Palmitate solution (0.5 mM) was freshly prepared before each experiment.

After 72 h, the cells were pre-incubated for 15 min in KRB and 0.5% BSA (pH 7.4) and were then incubated for 2 h with KRB containing 2 or 18 mM glucose and 0.5% BSA. Subsequently, as before, supernatants (300 µl) were collected, centrifuged and 200 µl were stored at −20°C for glucagon analysis. After the secretion study, the cells were washed once with 1 ml dH_2_O and the number of cells was estimated using nuclear staining with 0.01 mM SYTO 24 reagent (20 µl/well, Roche) and measured by FLUOstar Galaxy (BMG, Ramcon, Denmark). Glucagon levels were normalized to cell number.

### Glucagon content

Cells were exposed, for 72 h, to DMEM containing 18 mM glucose with or without 0.5 mM palmitate as well as ISV (10^−8^ or 10^−6^ M) in six-well plates as 3 ml/well. These culture conditions were used in all subsequent studies with the α-TC1–6 cell line, except where otherwise stated. The cells were washed once with 2 ml of cold PBS (GIBCO), replaced by 1 ml of glycine-BSA (glycine 100 mM, 0.25% BSA, pH 8.8; both Sigma) and were then transferred to 1.5 ml tubes. α-cells were disrupted by sonication (Branson Sonifier 250, Danbury, CT) on ice for 15 s (twice). After centrifugation at 16,000 rpm for 30 min, the supernatants were collected and stored at −20°C for glucagon and protein assay. Glucagon content levels were adjusted to protein concentration. Intracellular protein was measured by Bio-Rad detergent compatible protein assay kit (Bio-Rad Laboratories, Hercules, CA).

### Insulin assay

Insulin was analyzed by radioimmunoassay using guinea pig anti-porcine insulin antibody (Novo Nordisk, Bagsvaerd, Denmark) and mono-125I-(Tyr A14)-labeled human insulin (Novo Nordisk) as tracer and rat insulin as standard (Novo Nordisk). Bound and free radioactivity was separated by ethanol. The inter- and intra-assay variation coefficients were both less than 5%. ISV, like Stevioside and steviol at the concentrations studied, did not interfere with the insulin assay [46].

### Glucagon assay

Glucagon was analyzed by radioimmunoassay kit (Millipore Research Park Drive, St Charles, Missouri USA) according to manufacturer's instructions. The glucagon antibody is specific for pancreatic glucagon and has no cross-reaction with other islet polypeptides. The limit of sensitivity for the glucagon assay is 20 pg/mL.

### TG content

To determine TG content, cells were seeded in 24-well plates. After 72 h, the medium was removed, the cells were washed once with 1.0 ml of 0.9% NaCl, and the number of cells was estimated as above. The cells were frozen and stored for 1 h at −80°C. Subsequently, cells were incubated for 20 min with TG reagents (Roche; 250 µl/well). TG content was determined by a TG GPO-PAP kit (Roche) and was normalized to cell number. The recovery of TG content was ∼90%.

### ^3^H-thymidine incorporation in α-TC1–6 cells

^3^H-thymidine incorporation was used to monitor α-TC1–6 cells proliferation and DNA synthesis [47]. Briefly, cells (4×10^4^ cells/well) were seeded in 96-well plates (Wallac Oy, Turku) and were cultured in DMEM containing 18 mM glucose and 10% FBS. The next day, the medium was replaced with DMEM containing 5.5 mM glucose, 0.1% BSA and without FBS for 24 h (*i.e.* serum deprivation). The cells were then cultured for a further 72 h in DMEM containing 0.5% BSA, 18 mM glucose and 0.5 mM palmitate and 1 µCi [methyl-^3^H] thymidine (Amershan, Biosciences, UK). After 72 h, the cells were washed twice in cold PBS and then 200 µL liquid scintillator was added. The incorporated [methyl-^3^H] thymidine was counted by a 1450 MicroBeta TRILUX (Wallac, Turku, Finland).

### Isolation of RNA

The α-TC1–6 cells were cultured in 6-well plate for 72 h (as described in “Glucagon content”) prior to RNA extraction.

The MIN-6 cells (generously donated by Dr. Yamamoto, Kumamoto University school of Medicine, Japan) produce insulin and have morphological characteristics of pancreatic β-cells [31]. MIN6 cells between passages (38–40) were cultured separately in DMEM containing 25 mM glucose and 10% FBS at 5%/37°C. The cells were passaged weekly after trypsinization and replenished with fresh media. The cells are seeded in 6-well plates as 25% α-TC1–6 cells and 75% MIN6 cells; 100% α-TC1–6 cells; or 100% MIN6 cells.

Cells were then washed once with cold PBS and RNA was extracted from three different passages of cells using the AllPrep® RNA/Protein kit (Qiagen, Valencia, CA) according to the manufacturer's instructions. RNA was quantified by measuring absorbance at 260 and 280 nm (NanoDrop ND-8000 UV-Vis Spectrophotometer, NanoDrop Technologies Wilmington, DE). The integrity of the RNA was checked by visual inspection of the two ribosomal RNAs, 18S and 28S bands on a 1% non-denaturing agarose gel, stained with SYBR green.

### Real-time RT-PCR

Reverse transcription (RT): cDNA was synthesized, from 1 µg of total RNA for each 20 µl RT reaction, using the iScript™ cDNA synthesis Kit (Bio-Rad, Hercules, CA, USA) according to the manufacturer's instructions. cDNA was assayed undiluted for Acacb and diluted (1∶8) for Pax6, Pcsk2, Gcg, Insr, Irs1, Irs2, Acaca, Fasn, Srebf2, Pik3r1, Akt1 and Hypoxanthine guanine phosphoribosyl transferase (Hprt) in α-TC1–6 cells alone, (1∶100) for Ins1, Ins2, Gcg and Hprt in 25% α-TC1–6 and 75% MIN6 cells, both or just one or the other.

Real-time PCR (qPCR) was subsequently performed using TaqMan® assays using an ABI 7500 FAST machine (ABI, Foster City, CA, USA). Predesigned TaqMan® probes and primers were obtained from Applied Biosystems (ABI, Foster City, CA, USA) for Pax6 (assay Mm00443072_m1), Pcsk2(assay Mm00500981_m1), Gcg(assay Mm00801712_m1), Insr(assay Mm00439693_m1), Irs1(assay Mm01278327_m1), Irs2(assay Mm03038438_m1), Acaca(assay Mm01304277_m1), Acacb(assay Mm01204678_m1), Fasn(assay Mm00662319_m1), Srebf2(assay Mm01306289_m1), Pik3r1(assay Mm01282781_m1), Akt1(assay Mm01331624_m1), Ins1(assay Mm01259683_g1), Ins2(assay Mm00731595_gH) and Gcgr(assay Mm00433546_m1). Hprt(assay Mm01318743_m1) was assessed as endogenous control.

In brief, 10 µl reactions were prepared consisting of 5 µl 2× TaqMan ®FAST Universal Master Mix (P/N 4367846; ABI; Foster City, CA, USA), 0.5 µl 20×TaqMan Assay mix/probe (ABI; Foster City, CA, USA), 3.5 µl AcuGENE H_2_O and 1 µl cDNA (diluted or undiluted as above). Thermal FAST cycle programme was run at 95°C for 20 s; followed by 40 cycles at 95°C for 3 s and at 60°C for 30 s. Reactions were set up in duplicate for each sample and target gene expression levels were normalized to Hprt expression. All assays were performed in 96-well format plates (P/N 4366932) covered with optical adhesive cover (P/N 4311971; ABI; Foster City, CA). The TaqMan assay efficiencies were calculated to be in the range 89–108%. The 2^−ΔΔct^ method was applied to calculate the relative gene expression [48]. No template controls were included for each gene, as negative controls.

### Data and statistical analyses

Data is expressed as mean ± SEM of three or four independent experiments. Data analysis and graphs were performed using GraphPad Prism 4.0 (GraphPad Software Inc., San Diego, CA, USA). Statistical significance between two groups was evaluated by a two-tailed Student's unpaired T-test. One-way ANOVA followed by Dunnett's or Tukey *post hoc* tests (when appropriate) was used to detect statistically significant differences when more than two groups were compared. P<0.05 was considered to be significant.
